# Tetany in a Two-Year-Old Child With Undiagnosed Celiac Disease

**DOI:** 10.7759/cureus.86437

**Published:** 2025-06-20

**Authors:** Usha Ravi, Venkata Sushma Chamarthi, Rahul Kashyap, Ahmed Torky

**Affiliations:** 1 Department of Pediatrics, Pediatric Associates, Visalia, USA; 2 Department of Pediatrics, Valley Children’s Healthcare, Fresno, USA; 3 Department of Research, WellSpan Health, York, USA; 4 Department of Pediatric Endocrinology, Southern Illinois University School of Medicine, Springfield, USA

**Keywords:** celiac disease, hypocalcemia, hypomagnesemia, tetany, tingling

## Abstract

Celiac disease is an immune-mediated condition triggered by exposure to gluten in dietary products (such as wheat, rye, oats, and barley) in genetically predisposed individuals. It could present in different ways with classic symptoms of abdominal pain, diarrhea, bloating, or non-classic symptoms of anemia and neurological disturbances, including tetany. In rare cases, tetany can be an initial presentation of celiac disease. We report a case of a two-year-old child who presented with minimal gastrointestinal symptoms and finger pain and was eventually diagnosed with celiac disease. Interestingly, in this case, the patient remained symptom-free after implementing some dietary adjustments, supplements, and strict adherence to a gluten-free diet. Due to the asymptomatic and subtle nature of the disease, careful screening and detailed history-taking play a crucial role in facilitating prompt diagnosis and early treatment, thereby promoting a child's healthy lifestyle.

## Introduction

Celiac disease is an autoimmune T-cell-mediated disease in which the ingestion of gluten leads to the production of anti-tissue transglutaminase and anti-endomysial antibodies, as well as a marked T-cell response to gliadin in genetically predisposed individuals. Celiac disease is one of the most prevalent food-related illnesses in children, with a global prevalence of 1.4% [1]. Gluten is found in wheat, rye, barley, their crossbred grains, and possibly oats [2]. Classically, celiac disease in children presents with diarrhea, abdominal pain, bloating, vomiting, damage to tooth enamel, constipation, failure to thrive, headaches, Iron deficiency anemia, short stature, and weight loss [3]. Tetany is a rare presentation of celiac disease [4]. Small intestine epithelial cells and lamina propria T lymphocytes interfere in the inflammatory cascade, which leads to damage to the mucosa, which impairs the ability of the intestine to absorb nutrients and hence results in various lab abnormalities and symptoms [5]. Celiac disease could also present with electrolyte abnormalities such as hypocalcemia, hypomagnesemia, hypokalemia, and coagulopathy. Some probable causes of hypocalcemia in the pediatric population include rickets, severe vitamin D deficiency, hypomagnesemia, hypoparathyroidism, hyperphosphatemia, liver disease, sepsis, medication-induced, chronic kidney disease, pancreatitis, neonatal sepsis, maternal diabetes, maternal hypoparathyroidism, DiGeorge syndrome, and metabolic or mitochondrial disease [6].

## Case presentation

**Figure 1:**
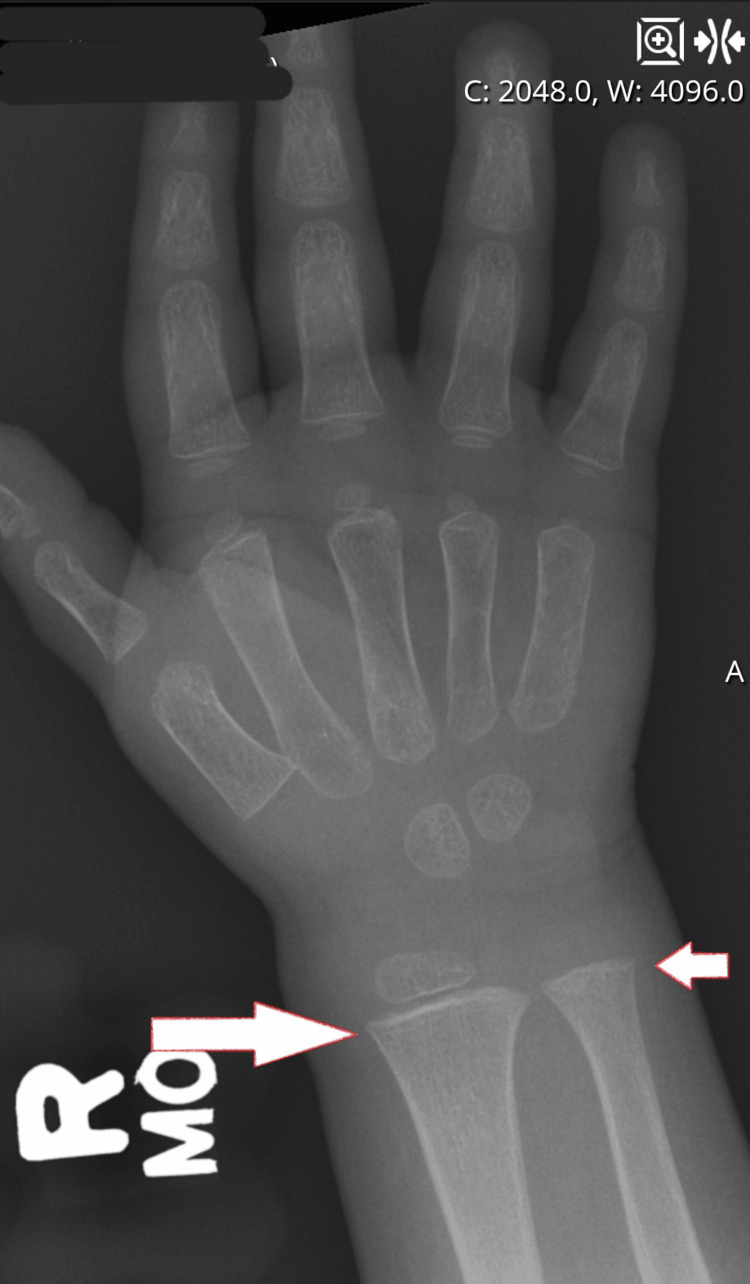
X-ray of the right wrist showing widened metaphases

A two-year-old healthy Caucasian boy with no significant past medical history was presented to a local Emergency Room (ER) with pain in all his fingers of the upper extremities. His growth at the time of presentation was reassuring, with weight around the 75th percentile and BMI around the 90th percentile. The patient's immunizations were up to date, and vitals were within normal limits. Parents reported that they started noticing facial twitching a week before presentation, and around the same time, he started having non-bloody diarrhea around three times per day. It was reported that diarrhea was followed by non-bilious, non-projectile emesis, which stopped on the day of presentation. Review of systems was otherwise negative. Physical exam was significant for positive Chvostek’s sign, and the patient had carpopedal spasm on measuring blood pressure, as well as fingers that looked like they were cramping. X-ray of the upper extremities on the right side showed widened metaphyses (Figure 1).

Some laboratory tests were done initially and showed hypomagnesemia and hypocalcemia (Table 1). 

**Table 1:** Laboratory values of the patient from presentation to discharge with normal ranges (normal ranges referenced from Southern Illinois University)

Labs	Initial presentation	s/p Calcium + Mag bolus	s/p Ca+Mag bolus	Started on vitamin D, oral Ca, oral Mag	Same meds	At discharge	Normal ranges
iCalicum	0.49	1.03	1.18	1.02	1.1	1.25	1.15-1.33 mmol/l
Calcium	5.4		7.8	7.6	8.1	8.9	8.5-10.1 mg/dl
Albumin				2.3		2.4	3.4-5.0 g/dl
Magnesium	0.7	1.1	2	1.4	2	2.1	1.6-2.6 mg/dl
Phosphorus	3.7		4.7	3	3.4	3.9	4.3-6.8 mg/dl
PTH (parathyroid hormone)	58			87.4	67.3		18.4-80.1 pg/ml
Alkaline phosphatase	117			130			104-345 IU/L
Vitamin D	<7						20-50 ng/ml
Potassium	2.8	2.4	3.2	3.5	4.4	4.1	3.5-5.1 meq/l
Sodium	143	142	140	143	143	140	136-146 meq/l
Ferritin					10.4		26-388 ng/ml
Celiac antibody (anti-tissue transglutaminase IgA)	>128						<7 u/ml
S. creatinine				<0.15			0.7-1.3 mg/dl
Urine calcium				<5			
Urine creatinine				22.3			
Urine magnesium				1.8			

Based on the laboratory values, he was given a total of 1.26 g of calcium gluconate IV bolus and a magnesium bolus and then was transferred to our hospital. The patient was admitted to the inpatient unit, and a further dietary history was obtained in terms of the amount of dairy consumption, different grains which the patient consumes, any other processed foods consumed by the patient, and any vitamins. It was found that his dairy consumption was approximately eight to 14 ounces of milk at daycare, up to four times per week, and occasional cheese intake. There was no history of yoghurt or any vitamin intake. He was seen by a primary care provider two months before presentation due to fatigue, and laboratory tests at that time showed a low ferritin level of 6 ng/mL (26-388 ng/ml), a calcium level of 8.9 mg/dL (8.5-10.1 mg/dl), and an alkaline phosphatase level of 103 IU/L (104-345 IU/L). Therefore, he was started on oral iron supplementation. An X-ray of the upper extremities was done, but did not show any evidence of rickets. His baseline ionized calcium level was 0.49, which improved to 1.03 (1.15-1.33 mmmol/L), but he continued to experience symptoms of finger pain; therefore, two additional calcium gluconate boluses (1 gram each) were administered. There was improvement in magnesium levels after the first bolus, but they were still low, so another magnesium bolus was administered. Later on, magnesium level increased to 2.0 mg/dl (1.6-2.6 mg/dl), and shortly after, PTH increased from 58 to 87.4 pg/ml (18.4-80.1 pg/ml).

**Figure 2:**
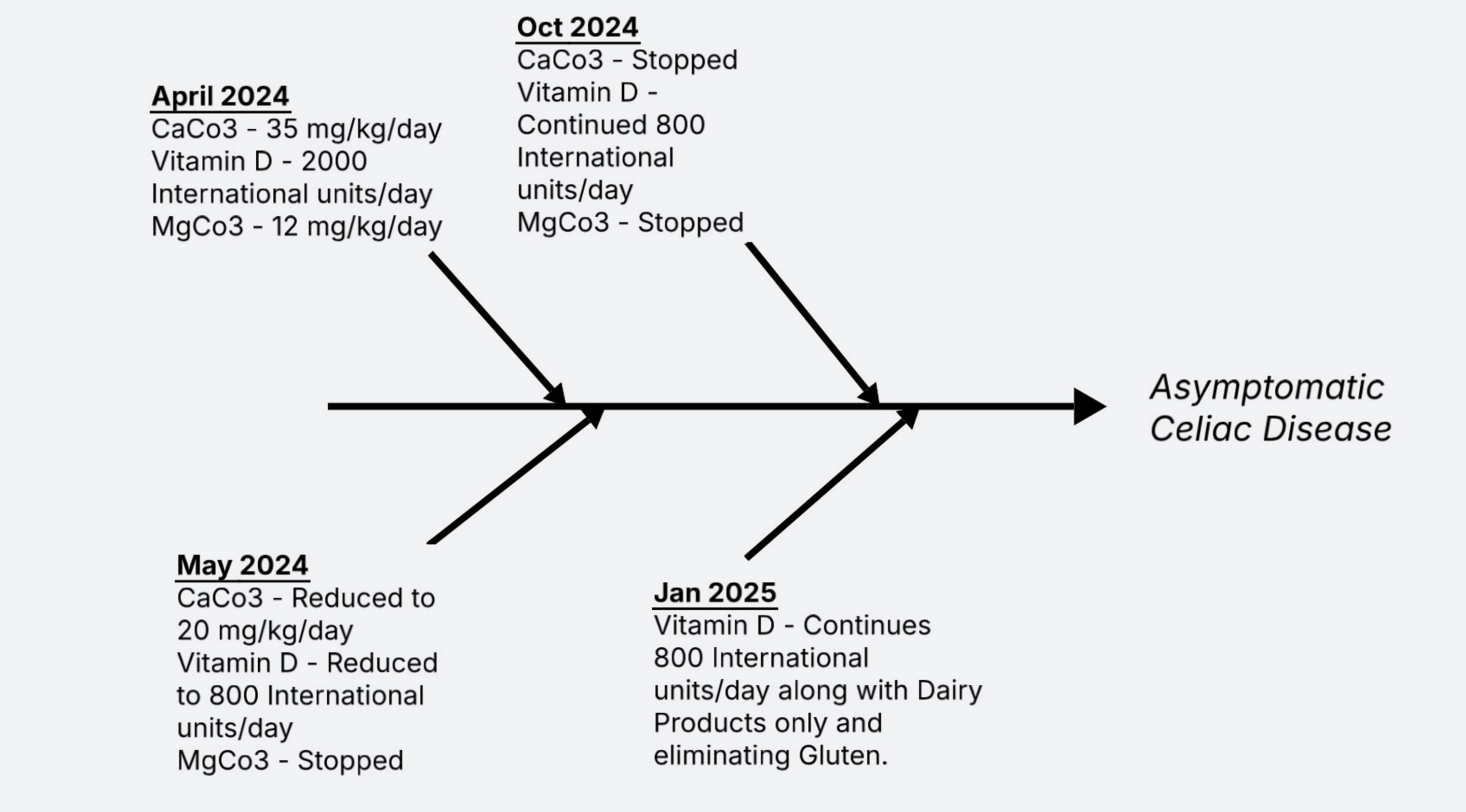
Patient’s clinical timeline from presentation to asymptomatic celiac disease

Medications were switched to oral medications: calcium carbonate 75 mg/kg/day (elemental), magnesium carbonate 12 mg/kg/day (elemental), and cholecalciferol 2000 units daily. Due to gastrointestinal (GI) symptoms, testing for celiac disease was done, which came back positive for tTG-IgA. Gluten elimination was started. Endoscopy was not performed because Pediatric Gastroenterology believed that there was sufficient evidence to support a diagnosis of celiac disease with presentation, lab values, and positive tTG-IgA in our patient, which was 10 times the upper limit of normal. A week after discharge [see Figure 2], the patient was on calcium carbonate 35 mg/kg/day (elemental) divided three times a day. He was also started on 2000 units of vitamin D daily and magnesium. In May 2024 (a month after discharge), he continued to be on the same amount of calcium carbonate and a similar amount of vitamin D and magnesium daily and did not have any symptoms (Table 1).

Based on the levels, vitamin D was reduced to 800 units a day, magnesium was stopped, and calcium was reduced to 20 mg/kg/day (elemental), and in addition to that, dietary dairy intake was increased. A dietitian was consulted and helped with education about eliminating gluten-containing foods. In October 2024 (six months after discharge), his father reported that the patient started having normal bowel movements and felt more energetic with no signs of jitteriness, cramping, or lethargy. His parents were instructed to discontinue the calcium but continue vitamin D 800 units. Most recently, in January 2025 (nine months after discharge), he continues to be on dairy products as the only source of calcium, takes 800 units of vitamin D, and does not take any magnesium supplements, all while eliminating gluten (Figure 2).

## Discussion

Hypomagnesemia is an uncommon cause of hypocalcemia. Very low magnesium levels can cause hypocalcemia by PTH resistance or by preventing PTH release. Serum calcium levels regulate the secretion of parathyroid hormone from the parathyroid gland, but serum magnesium levels could do the same. Low serum magnesium levels can stimulate the secretion of parathyroid hormone, but very low magnesium levels could block the secretion. This block could cause severe hypocalcemia in severely hypomagnesemic patients. Activation of the alpha-subunit of heterotrimeric G-protein causes this effect. This activation mimics the activation of the calcium-sensing receptor, which inhibits the secretion of parathyroid hormone [7]. The parathyroid gland will detect hypocalcemia via receptors in the membrane and rapidly release parathyroid hormone. Once PTH is released, it facilitates net bone formation over bone resorption, increases urinary calcium retention, and causes renal activation of 1,25-dihydroxyvitamin D, whose role is to increase intestinal calcium resorption. As the calcium level normalizes, negative feedback is sent to stop the release of PTH [8].

Celiac disease can present at any age and usually presents with a wide range of symptoms, ranging from asymptomatic presentation diagnosed through screening tests, classic intestinal symptoms of diarrhea, abdominal pain, and weight loss, to non-classic extraintestinal symptoms of osteoporosis, anemia, and neurological disturbances. Sometimes, patients could present with symptoms because of nutrient deficiencies of iron, folate, vitamins B12 or D, calcium, magnesium, or zinc. Celiac disease causes malabsorption, resulting in many macro- and micronutrient deficiencies, including magnesium. Besides malabsorption, a low-magnesium diet could also contribute to the disease, and magnesium deficiency could persist even after a gluten-free diet, highlighting the importance of encouraging a diet rich in magnesium [9]. Tetany is a nerve hyperexcitability disorder characterized by symptoms that can range from paresthesia, tingling of the hands and feet, to spasm of the muscles in the wrists and ankles (carpopedal spasm), laryngospasm, numbness, and brief, recurrent convulsions. Tetany can result from hypocalcemia, hypomagnesemia, or alkalosis in the interstitial tissue fluid [10]. The European Society for Paediatric Gastroenterology, Hepatology and Nutrition (ESPGHAN) guidelines from 2012 recommend testing for the tTG-IgA, which is highly sensitive and specific as the initial screening test for diagnosing suspected celiac disease, and the total IgA to rule out selective IgA deficiency. If the tTG-IgA is higher than 10 times the upper limit of normal (ULN) in a symptomatic patient like ours, it should be discussed with parents to make a diagnosis without biopsy. If tTG-IgA is found as positive (lower than 10 times ULN), then gastroduodenscopy and multiple biopsies of the small intestine should be performed to confirm the diagnosis [11].

The unique feature is that our patient had reassuring growth with mild GI symptoms, which started only a few days before the presentation. We found a case report in which a child presented similarly with Tetany, hypomagnesemia, and hypocalcemia but also had steatorrhea and weight loss [12]. With a short duration of diarrhea, which had resolved by the day of presentation, our patient presented with Tetany. This case is a reminder to have a very high index of suspicion for celiac disease, even with mild symptoms. As the patient remained asymptomatic with a gluten-free diet, it significantly underscores various subtle presentations of celiac disease and adds to the existing literature.

## Conclusions

Celiac disease causes damage to the intestine in genetically predisposed individuals, and the presentations vary widely. No genetic testing was done in our patient. If the Tg-IgA is positive (>10 ULN), HLA DQ2/8 analysis is not recommended in the ESPGHAN guidelines 2020. Due to the variability in presentation, it becomes hard for a clinician to diagnose celiac disease, and hence, all causes of unexplained diarrhea, weight loss, anemia, short stature, enamel issues, and laboratory abnormalities need to be screened for celiac disease. Celiac disease should be investigated in patients with a high risk of celiac disease, like type 1 DM, Down’s syndrome, autoimmune thyroid disease, Turner’s syndrome, selective IgA deficiency, autoimmune liver disease, and first-degree relatives of celiac disease, even if they are asymptomatic. Hypomagnesemia is an uncommon cause of hypocalcemia and is mostly caused by magnesium loss through the gut or the kidneys. This case highlights the rare combination of celiac disease and hypomagnesemia that is severe enough to cause tetany. A high index of suspicion and detailed evaluation of all causes of hypocalcemia is imperative for proper management. Very careful history taking, especially focusing on detailed nutrition history, even in the absence of weight loss, needs to be looked further into with caution, highlighting the importance of assessing all possibilities. It also emphasizes the role of a dietitian in educating families about gluten-containing foods and adds this unique presentation to the existing literature.
